# Beach Bug Bingo: Toward Better Prediction of Swimming-Related Health Effects

**Published:** 2006-01

**Authors:** Nancy Bazilchuk

Swimming is a popular pastime in the United States. The 2000–2002 National Survey on Recreation and the Environment reported that each year an estimated 89 million Americans swim in recreational waters including lakes, oceans, streams, rivers, and ponds. But swimming waters may also be contaminated by human sewage from treatment plants and runoff, raising the risk of gastrointestinal (GI) illness in swimmers. The recommended test for measuring contamination requires culturing fecal indicator bacteria, which means that beach managers must wait 24 hours for results. This built-in delay is problematic, potentially exposing swimmers to unhealthy water quality and sometimes resulting in unnecessary beach closures. Now a team of federal researchers has shown that a rapid method for measuring water quality can accurately predict swimming-related health effects **[*EHP* 114:24–28]**.

The researchers conducted health surveys of beachgoers at two public beaches, one on Lake Michigan and one on Lake Erie, and compared them with thrice-daily water quality measurements along transects at the beaches. They evaluated water quality using a modified version of the polymerase chain reaction method (QPCR) to quantify indicator bacteria in water samples. The advantage of this method is that it can provide results in two hours or less. The researchers chose *Enterococci* and *Bacteroides* as their indicator organisms.

Survey participants were interviewed as they left the beach; follow-up interviews were conducted by telephone 10 to 12 days after the beach visit. When researchers compared results of the water quality tests to participant reports of GI and other illnesses, they found a significant trend between increased reports of GI illnesses and *Enterococci* at the Lake Michigan beach and a positive, though statistically insignificant, trend for *Enterococci* at the Lake Erie beach. *Bacteroides* did not prove to be as powerful in predicting illness, with an insignificant positive trend found only at the Lake Erie beach and no trend at the Lake Michigan beach.

When results from the two beaches were combined, the trend for *Enterococci* and GI illness remained statistically significant, a finding that held true even when samples collected at 8:00 a.m. were compared to daily averages. Beach managers could thus test early-morning samples to assess water quality and, if necessary, close beaches before the majority of swimmers were exposed.

In spite of the promising nature of the findings, the authors caution that much research remains to be done before the results can be generalized. One of the key remaining questions relates to the method itself: QPCR relies solely on the presence of DNA to quantify organisms, so pathogens are detected even if they are dead and thus harmless. QPCR may therefore suggest a problem with the water when in fact there is none. The authors say additional studies should help determine if the approach is robust enough to be used in water quality regulations.

## Figures and Tables

**Figure:**
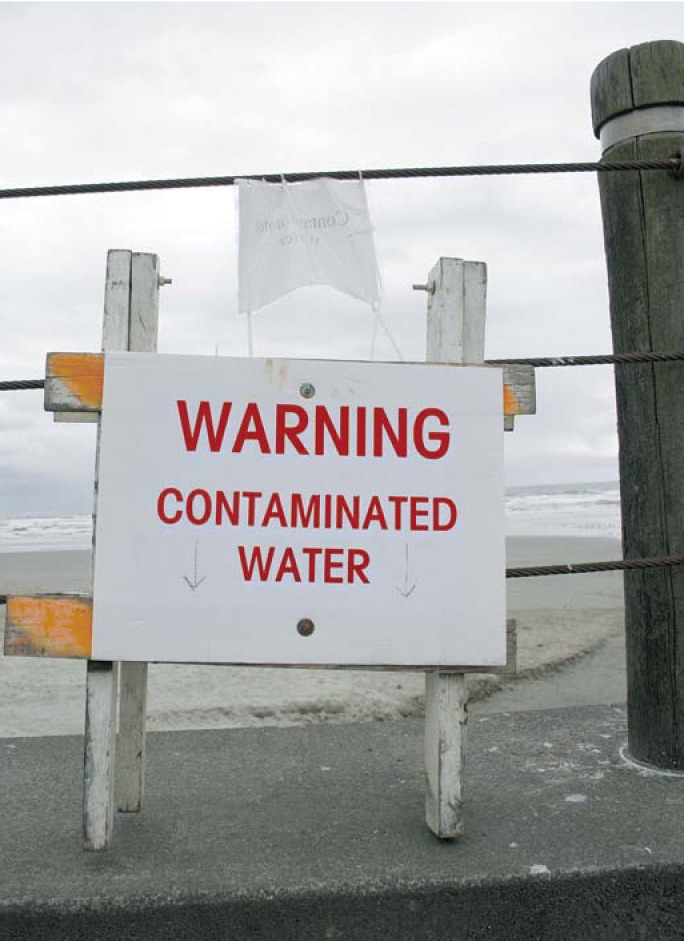
Wave of the future? If validated, a modified polymerase chain reaction method may become useful for earlier identification of hazardous beach water conditions.

